# Successful access to the ampulla for endoscopic retrograde cholangiopancreatography in patients with situs inversus totalis: a case report

**DOI:** 10.1186/s12893-017-0307-x

**Published:** 2017-11-28

**Authors:** Jung Min Lee, Jae Min Lee, Jong Jin Hyun, Hyuk Soon Choi, Eun Sun Kim, Bora Keum, Yoon Tae Jeen, Hoon Jai Chun, Hong Sik Lee, Chang Duck Kim

**Affiliations:** Division of Gastroenterology and Hepatology, Department of Internal Medicine, Korea University College of Medicine, Seoul, South Korea

**Keywords:** Situs inversus, Endoscopic retrograde cholangiopancreatography, Choledocholithiasis

## Abstract

**Background:**

Although various endoscopic techniques in situs inversus have been reported, endoscopic retrograde cholangiopancreatography (ERCP) in patients with situs inversus is always challenging even for an experienced endoscopist. We performed ERCP using two different techniques, and compare the merits of each technique.

**Case presentation:**

A 74-year-old woman presented with epigastric pain and jaundice for 3 days. Computed tomography revealed diffuse dilatation of the biliary tree, with multiple intrahepatic duct and common bile duct (CBD) stones, in addition to situs inversus totalis. ERCP was performed twice for CBD stone to remove the CBD stones using two techniques. For the first technique used, the patient was placed in a prone position with the endoscopist on the right side of the table. First, the endoscope was rotated 180° counterclockwise in the stomach, and was then shortened by turning 180° the counterclockwise again in the duodenum. For the second technique, we assessed the second portion of the duodenum by following the lesser curvature, while slowly turning the endoscope clockwise.

**Conclusion:**

We present an unusual case of biliary stones in a patient with situs inversus who was treated using modified ERCP techniques.

**Electronic supplementary material:**

The online version of this article (10.1186/s12893-017-0307-x) contains supplementary material, which is available to authorized users.

## Background

Situs inversus is a very rare anomaly in which the internal organs are reversed, as if in a mirror image of their normal position [1]. The incidence of this autosomal recessive condition is approximately 1 in 20,000 [2]. The anatomy of the left and right sides is reversed; hence, it is more difficult to perform endoscopic retrograde cholangiopancreatography (ERCP) and sphincterotomy in patients with situs inversus than in normal patients [3, 4]. Although the patient lies prone and the endoscopist performs ERCP on the right side of the table, as in normal patients, several techniques have been used in situs inversus to increase the success rate [4–6]. Among these, the “twist” method, the endoscope is rotated 180° clockwise in the stomach and subsequently rotated 180° clockwise after entering the duodenum. However, in our experience, it is possible to perform ERCP in a patient with situs inversus without using the twist method.

We present an unusual case in which ERCP was performed using two different techniques in the same situs inversus patient, and compare the advantages and disadvantages of each technique.

## Case presentation

**Fig. 1:**
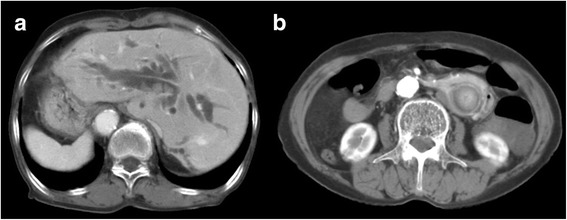
**a** Diffuse dilatation of biliary tree with multiple intrahepatic duct stones and CBD stones. **b** CT revealed situs inversus totalis

**Fig. 2:**
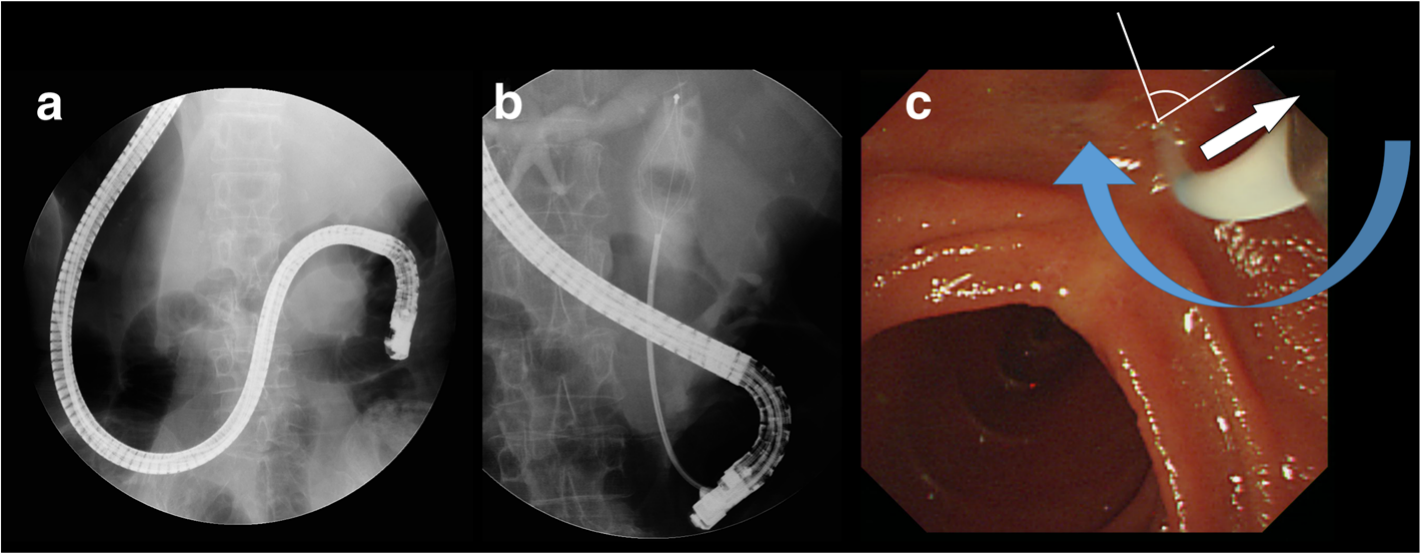
**a**, **b** The endoscope rotate in 180° counterclockwise in the stomach, and shortened by again rotating 180° to the counterclockwise in the duodenum. **c** In the endoscopic view, the ampulla was deviated to the right and right-sided direction The bile duct direction (*white arrow*), catheter device direction (*blue arrow*), and angle between two vectors (*white line*) are indicated



A 74-year-old woman presented with epigastric pain and jaundice for 3 days. She had epigastric tenderness but a negative Murphy’s sign. The aspartate transaminase level was elevated to 191 IU/L, the alanine transaminase level to 79 IU/L, and total bilirubin level to 15.23 mg/dL. The alkaline phosphatase level was 409 IU/L and γ-glutamyltransferase level was 815 IU/L. Computed tomography (CT) revealed diffuse dilatation of biliary tree, with multiple intrahepatic duct stones and common bile duct (CBD) stones (Fig. 1a). Previously undiagnosed situs inversus viscerum was also found on CT (Fig. 1b). Before performing ERCP, an endoscopic examination using conventional gastroscopy was performed, which showed a reversed anatomy of the gastrointestinal tract. For removal of bile duct stones, the patient was placed in the prone position and the endoscopists performed ERCP from the right side of the table. The endoscope was rotated 180° counterclockwise in the stomach. After entering the duodenum, the endoscope was again shortened using a 180° counterclockwise rotation (Fig. 2a,b, Additional file 1 and Additional file 2). Although access was relatively easy, the ampulla in the endoscopic view was deviated to right side and right-upward direction (Fig. 2c). Moreover, it was difficult to control the endoscope owing to the looped endoscope shaft. In the first ERCP, the cholangiogram revealed a large filling defect and diffuse dilatation of tge CBD. After performing a sphinterotomy and mechanical lithotripsy, we removed a bile duct stone. However, several stones still remained in the CBD on follow-up cholangiography.

**Additional file 1:** Video for counterclockwise-counterclockwise rotation. (AVI 5645 kb)


**Fig. 3:**
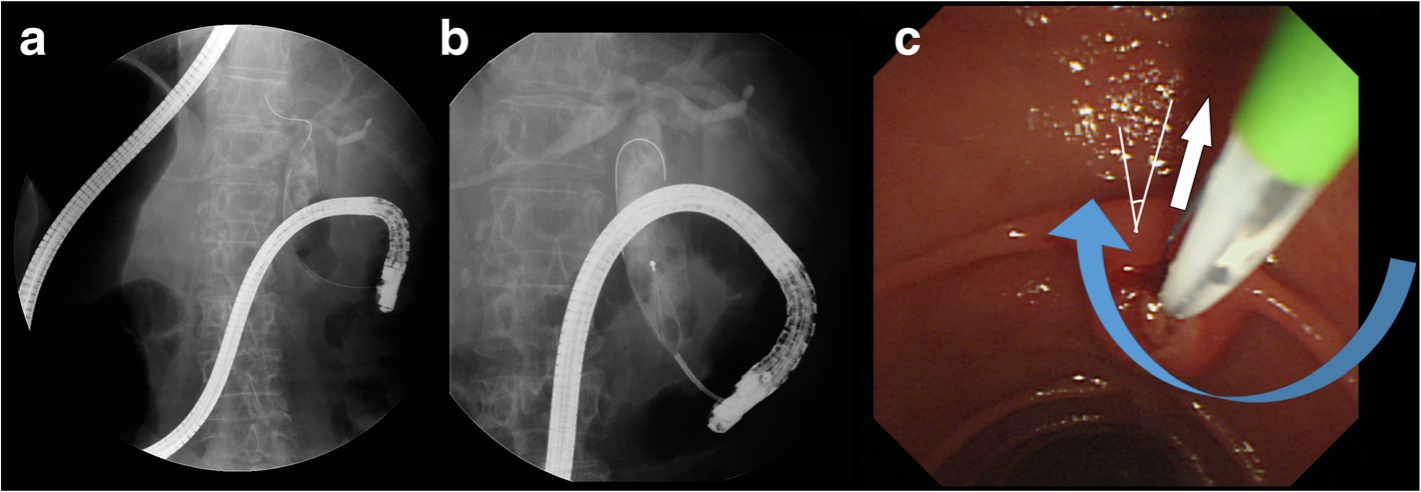
**a**, **b** The endoscope reached the second portion of the duodenum by following the lesser curvature, while slowly rotating the endoscope clockwise. **c** In endoscopic view, the ampulla was shown at the center and upward direction. The bile duct direction (white arrow), catheter device direction (blue arrow), and angle between two vectors (white line) are indicated



Two days later, we performed a second ERCP using a different method. The patient was again placed in a prone position with the endoscopist on the right side of the table. This time, the second portion of the duodenum was reached by following the lesser curvature, while slowly rotating the endoscope clockwise (Fig. 3a,b, Additional file 3 and Additional file 4). Despite the reversed organ anatomy, the endoscope was successfully advanced to the second portion of the duodenum using a slow and careful technique. In this approach, there was no looped endoscope shaft. The ampulla appeared at the center and upward direction of the endoscopic view screen (Fig. 3c). Although endoscopic access to the duodenum was more difficult than during the first ERCP using a counterclockwise rotation, selective CBD cannulation and stone removal were easier, owing to correct location of the orifice and direction of the bile duct.

**Additional file 3:** Video for counterclockwise-clockwise rotation. (AVI 4063 kb)


The patient’s gallstones were completely removed with the second ERCP, and her serum total bilirubin levels decreased to 1.83 mg/dL. There were no complications during the procedure, and the patient was discharged in good condition.

## Discussion

Although widely used in patients with biliary tract disease, ERCP procedures are challenging in the presence of anatomical abnormalities and even a skilled endoscopist can encounter technical difficulties when performing ERCP in patients with situs inversus. There have been only a few reports on ERCP in patients with situs inversus [2–6].

To increase the success rate, several techniques have been introduced for ERCP in patients with situs inversus [5]. In one technique, the endoscope is rotated 180° clockwise in the stomach; after entering the duodenum, the endoscope is again rotated 180° clockwise [7]. A similar technique involves turning the duodenoscope 180° clockwise in the stomach and using a rotating sphincterotome for cannulation [8]. These methods are performed in a familiar environment, as the position of the patient, the endoscopist, the endoscope machine, and the monitor are the same as usual. However, the techniques require a skilled endoscopist with abundant experience. In addition, if a loop is formed, it may be difficult to position the endoscope at the ampulla.

Another option is a “mirror image” method. The patient is usually placed in the right prone position because of the reversal of internal organs, and the endoscopist performs the procedure from the left side of the table. However, shortening the endoscope using a counterclockwise rotation in the mirror image method is inconvenient, as the endoscopist is required to manipulate the endoscope with the right hand. In addition, the position of the patient, endoscopist, the endoscopic machine, and the monitor must be changed. Alternatively, “inversed normal” ERCP can be performed with the patient in the prone position and the endoscopist on the right side of the table. The endoscope is advanced to the duodenum by following the lesser curvature without rotating the endoscope. In addition, when there is axis malalignment, endoscopic papillary balloon dilation (EPBD) can be relatively easy to perform once deep cannulation is accomplished. EPBD with or without endoscopic sphincterotomy (EST) is an alternative method to EST alone for stone removal.

We present an unusual case of CBD stones in a patient with situs inversus. Successful ERCP was performed using two different techniques. Specifically, we used two different modified twist methods. In the first ERCP, counterclockwise rotation was used to insert the endoscope with relative ease. The endoscope was fully rotated once. However, cannulation was difficult because the ampulla was on the right side of the screen. In the second ERCP with clockwise rotation, a second rotation was not performed. Cannulation was easily performed, as the ampulla was located at the center of the screen, slightly to the left. However, insertion of the endoscope was relatively difficult. The methods have not standardized, but show that an appropriate technique can be selected according to the patient’s position, loop formation and air volume.

## Conclusion

We report a case requiring repeat ERCP, in which a skilled endoscopist used two different techniques in the same patient, with the patient’s in the same position both times. A prospective study is required to determine the optimal approach in situs inversus.

## Additional files

Additional file 2: Figure for counterclockwise-counterclockwise rotation. (JPEG 75 kb)

Additional file 4: Figure for counterclockwise-clockwise rotation. (JPEG 76 kb)

## Abbreviations

CBD: Common bile duct
CT: Computed tomography
ERCP: Endoscopic retrograde cholangiopancreatography
EST: Endoscopic sphincterotomy
