# Supplementary figures and images for: Successful access to the ampulla for endoscopic retrograde cholangiopancreatography in patients with situs inversus totalis: a case report
Source: BMC Surg. 2017 Nov 28;17:112. doi: 10.1186/s12893-017-0307-x (PMC5706404; doi:10.1186/s12893-017-0307-x)

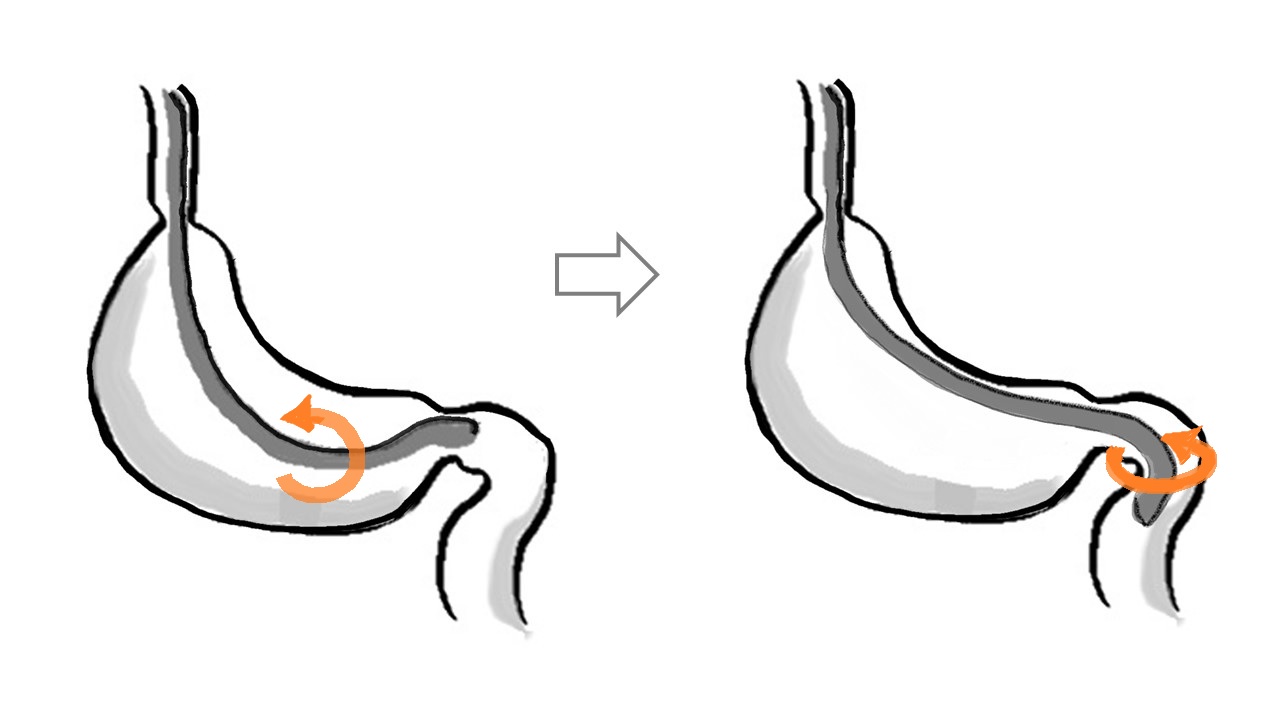

Supplement: Supplementary file 3 — Figure for counterclockwise-counterclockwise rotation. (JPEG 75 kb) [file 12893_2017_307_MOESM2_ESM.jpg]

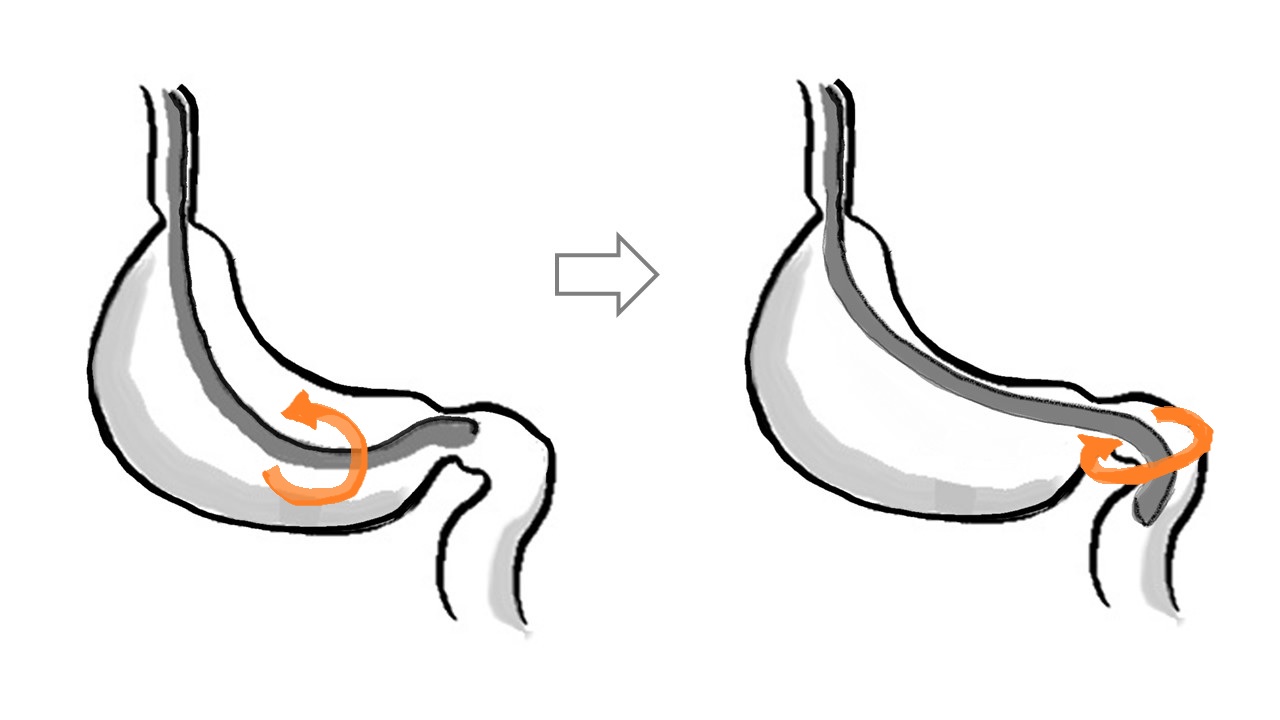

Supplement: Supplementary file 4 — Figure for counterclockwise-clockwise rotation. (JPEG 76 kb) [file 12893_2017_307_MOESM4_ESM.jpg]
